# Editorial: Medication safety and interventions to reduce patient harm in low- and middle-income countries

**DOI:** 10.3389/fphar.2022.1124371

**Published:** 2023-01-09

**Authors:** Mansour Adam Mahmoud, Johanna C. Meyer, Ahmed Awaisu, Joseph Fadare, Ahmed Ibrahim Fathelrahman, Fahad Saleem, Hisham Aljadhey, Brian Godman

**Affiliations:** ^1^ Department of Clinical and Hospital Pharmacy, Taibah University, Medina, Saudi Arabia; ^2^ Department of Public Health Pharmacy and Management, School of Pharmacy, Sefako Makgatho Health Sciences University, Pretoria, South Africa; ^3^ South African Vaccination and Immunisation Centre, Sefako Makgatho Health Sciences University, Pretoria, South Africa; ^4^ College of Pharmacy, Qatar University, Doha, Qatar; ^5^ Department of Pharmacology and Therapeutics, Ekiti State University, Ado Ekiti, Nigeria; ^6^ Department of Medicine, Ekiti State University Teaching Hospital, Ado Ekiti, Nigeria; ^7^ Department of Clinical Pharmacy, College of Pharmacy, Taif University, Taif, Saudi Arabia; ^8^ University of Balochistan, Quetta, Pakistan; ^9^ Saudi Food and Drug Authority, Riyadh, Saudi Arabia; ^10^ Centre of Medical and Bio-Allied Health Sciences Research, Ajman University, Ajman, United Arab Emirates; ^11^ Department of Pharmacoepidemiology, Strathclyde Institute of Pharmacy and Biomedical Sciences, University of Strathclyde, Glasgow, United Kingdom

**Keywords:** medication safety, patient harm, adverse drug events (ADE), medication error, adverse drug reaction (ADR), interventions

The safe and rational use of medicines is crucial, especially from the context of low- and middle-income countries (LMICs) where medicine spending accounts for a considerable proportion of healthcare costs, and much of this is out-of-pocket expenditure (Cameron et al., 2009; Ofori-Asenso and Agyeman, 2016). Consequently, medicines should not be over-used or misused as seen with antibiotics in ambulatory care among patients with self-limiting illnesses or in hospitals for patients with COVID-19 (Godman et al., 2020; Langford et al., 2021), as this increases antimicrobial resistance (AMR) with its considerable impact on morbidity, mortality, and cost (Hofer, 2019; Antimicrobial Resistance Collaborators, 2022; GBD, 2023). Similarly, efforts are needed to improve medicine use in patients with chronic non-communicable diseases, including enhancing adherence to prescribed medicines, to improve outcomes and reduce complications (Kirk et al., 2017; Rezende Macedo do Nascimento et al., 2020; Chan et al., 2021; Nowak et al., 2022). Alongside this, reducing the potential for drug-drug interactions (DDIs) especially among patients with multiple co-morbidities. Furthermore, increased knowledge about possible adverse drug events (ADE) can reduce medication errors and adverse drug reactions (ADRs), with their substantial impact on morbidity, mortality and cost (Chan et al., 2016; Mouton et al., 2016; Formica et al., 2018). However, major issues with medication safety, including ADRs and medication errors, are severely hampered by inadequate patient education and counselling, low health literacy and considerable under reporting of ADRs (Mahmoud et al., 2014), with issues of medication misadventure more prevalent in LMICs. It was against this background, that the need for this Research Topic was identified, which resulted in 19 original research papers. It is hoped that this collection of original papers will provide future guidance to reduce patient harm, improve the care of patients and their quality-of-life.

ADR reporting is an issue across countries, especially in the ambulatory care setting (Ampadu et al., 2016; Gidey et al., 2020; Haines et al., 2020; Sefah et al., 2021; Mahmoud et al., 2022). This was identified by Karuppannan et al. with many pharmacists, especially community pharmacists, not reporting ADRs even when identifying them (Karuppannan et al.). However, it was reassuring to note the study by Jiang and colleagues, in which they documented the extent of current ADRs in their hospital over a 10-year period, stratified according to the severity of the ADRs (Jiang et al.). In addition, the extent of ADRs caused by DDIs was similarly reported. The investigators concluded that increased training can assist physicians with their knowledge of ADRs and associated DDIs to improve patient safety and care outcomes (Jiang et al.). Alsheikh and Alasmari also found that community pharmacists in Saudi Arabia were knowledgeable about ADRs. Furthermore, they had good attitude and practices concerning pharmacovigilance and ADR reporting (Alsheikh and Alasmari), which is encouraging. These findings contrast with those of Hu et al. in China, who found that whilst hospital pharmacists typically had a positive attitude towards ADR reporting, there were concerns with their actual knowledge and practices (Hu et al.). This is a concern since hospital pharmacists are key role players in LMICs, educating physicians regarding the importance of monitoring and reporting of ADRs to improve patient care and safety (Terblanche et al., 2018).

In their study, Yang and co-authors showed a positive impact of drug and therapeutic committees (DTCs) in hospitals on reducing prescribing errors and inappropriate prescribing of antibiotics as well as associated AMR through antimicrobial stewardship (AMS) activities, alongside reducing costs (Yang et al.). This is important given concerns with currently a limited number of active DTCs and their impact across LMICs, including encouraging ADR reporting and improving antimicrobial use through AMS activities, due to resource constraints, limited training and other issues; however, this is changing (Cox et al., 2017; Matlala et al., 2017; Fadare et al., 2018; Siachalinga et al., 2022).

Trained community pharmacists can also play a key role in reducing unnecessary purchasing of antibiotics without a prescription, especially for self-limiting conditions such as acute respiratory infections (Marković-Peković et al., 2017; Mukokinya et al., 2018). This is an issue in countries and regions such as post-conflict zones in Pakistan where there is currently poor knowledge, attitude and practices among citizens towards antibiotics and AMR (Khan et al.). Previous studies have demonstrated high rates of purchasing of antibiotics without a prescription in Pakistan, including ‘reserve’ antibiotics as per the WHO AWaRe classification (Sharland et al., 2018), which needs to be urgently addressed as part of national action plans, if Pakistan is to achieve its desired goals (Saleem et al., 2018; Atif et al., 2019; Saleem et al., 2020). In the case of children, pictorial storybook telling can assist with enhancing their knowledge regarding the rational use of medicines, including antibiotics (Bakaruddin et al.), which is a consideration for the future. In a number of LMICs, especially among African and Asian countries, such activities are needed to address rising AMR and its consequences, including increasing the use of ‘Watch’ antibiotics (Klein et al., 2021; Antimicrobial Resistance Collaborators, 2022).

The timely identification of risk factors associated with ADEs is also important to improve future patient care. In their study, Khan et al. found that the prescribing of bedaquiline alongside other active treatments lowered the chance of ADEs in patients with multidrug-resistant *tuberculosis* (TB) (Khan et al.). Alongside this, elderly patients, active smokers and those experiencing a delay in treatment were more prone to ADEs. The care of TB patients can also be improved through information provided regarding the rational use of medicines, early detection and management of ADEs as well as general counselling from clinical pharmacists (Khan et al.).

Improving the prescribing of medicines to treat cardiovascular disease in the elderly to reduce potentially inappropriate prescribing (PIP) in LMICs, and their associated consequences, is becoming critical with growing prevalence and mortality rates (WHO, 2021). Xingwei and colleagues discuss the development of a learning-based risk warning model to aid physicians in identifying key factors in this population that could result in PIP to provide future guidance (Xingwei et al.). In their study, Očovská et al. highlighted the importance of both effectiveness and safety when treating patients to help reduce drug-related hospital admissions (DRA) (Očovská et al.). This is especially the case with diuretics and antithrombotic medicines which are both effective; however, both are among the most common classes of medicines causing DRA (Očovská et al.).

Conducting research to identify ways to improve adherence to medicines in patients with long-term diseases is also important. This is especially the case during pandemics with their impact on clinic closures and associated concerns with the subsequent monitoring of patients (Kluge et al., 2020). Ahmed and colleagues identified key enablers to enhance adherence to prescribed medicines in patients with HIV/AIDS to assist with this (Ahmed et al.). They also identified key barriers to adherence, which included lack of social support, stigma and COVID-19 related lockdown measures (Ahmed et al.), which need to be addressed going forward.

In their study, Liu et al. demonstrated the considerable concerns regarding the management of patients with presumptive asthma among primary care providers in rural China (Liu et al.). In their vignette, only 10% of providers prescribed the correct medicines, whilst 65% prescribed antibiotics, which were considered unnecessary. Furthermore there was high use of injections, which was also unnecessary among asthma patients, calling for a considerable re-think of incentives and educational approaches to improve the future care of these patients (Liu et al.). Sharing of medicines is also a problem across countries, including unused medicines left over from a course of treatment (Mahlaba et al., 2022), as this can delay diagnoses, enhance DDIs and ADRs as well as AMR with antibiotics (Song et al.). The authors showed this was a considerable problem in South Korea, which calls for greater public education campaigns similar to other countries (Song et al.).

On a positive note, Yi et al. demonstrated that the introduction of collaborative pharmaceutical care services among patients with Parkinson’s disease in China can reduce drug-related problems as well as improve patients’ medication regimens, including dosage adjustments where needed, and adherence thereby improving their quality-of-life (Yi et al.). Consequently, providing a rationale for further improving pharmacy services across China and other LMICs with ageing populations.

Some papers in this Research Topic also focussed on very specific issues. For instance, Bibi and colleagues found in their observational cohort study that biodegradable polymer drug-eluting stents had comparable clinical outcomes to durable polymer stents when used for primary percutaneous coronary interventions (Bibi et al.). Studies such as this will assist policymakers and clinicians in their decision-making, especially in resource-constrained settings. In their study, Zhang et al. were concerned that the prescribing of urate-lowering-therapy (ULT) would adversely influence the progression of kidney function in patients with asymptomatic hyperuricemia. Encouragingly, they found that ULT did not delay the progression of kidney function; although further studies are needed (Zhang et al.). Chai and associates were concerned that the increasing use of dipeptidyl peptidase-4 inhibitors, glucagon-like peptide-1 receptor agonists or sodium-glucose cotransporter-2 inhibitors in patients would increase the risk of fractures among patients with type 2 diabetes (Chai et al.). This is an issue with increasing rates of diabetes globally combined with a growing prevalence of complications in sub-optimally controlled patients (Chan et al., 2021). Nevertheless, the authors believed an association was unlikely based on their network meta-analysis (Chai et al.).

Mushtaq and co-authors were concerned with the emergence of resistant strains in patients with hepatitis C virus infection, despite the effectiveness of direct-acting antivirals (DAAs) (Mushtaq et al.). Based on their findings, they advocated that direct resistance testing should be encouraged in the future to optimise re-treatment strategies in patients failing on DAA therapy, given the importance of effectively treating these patients (Mushtaq et al.). This is likely to be followed up in the future. Finally, Mei et al. found that Nao-Xue-Shu, a traditional Chinese medicine, combined with nifedipine showed improved effectiveness in patients with hypertensive intracerebral haemorrhage compared with the other combinations, and Nao-Xue-Shu combined with nimodipine may be more effective in reducing proinflammatory factor expression in these patients (Mei et al.).

In conclusion, there were a considerable number of papers in this Research Topic. Strengthening pharmacovigilance policies and standards in LMICs is crucial to increase ADR reporting and improve patient safety. A continuous development program among healthcare professionals concerning pharmacovigilance along with participation in advocacy for ADR reporting are both key to improving pharmacovigilance in practice. There is certainly a need to reduce DDIs and associated AMRs, to improve future patient care and reduce healthcare costs. Community and hospital pharmacists, as well as physicians, have a key role to play to encourage further reporting to improve patient care. Improving adherence to medicines is also a key area for the future, alongside the potential for collaborative pharmaceutical care services. Finally, concerted efforts are needed to improve appropriate prescribing and dispensing of antibiotics across sectors in an attempt to curb the menace of AMR. Antimicrobial stewardship programmes are key in this respect.
